# Factors affecting driver injury severity in fatigue and drowsiness accidents: a data mining framework

**DOI:** 10.5249/jivr.v14i1.1679

**Published:** 2022-01

**Authors:** Ali Tavakoli Kashani, Marzieh Rakhshani Moghadam, Saeideh Amirifar

**Affiliations:** ^*a*^ School of Civil Engineering, Iran University of Science & Technology, Tehran, Iran.; ^*b*^ Road Safety Research Center, Iran University of Science & Technology, Tehran, Iran.

**Keywords:** Fatigue and drowsiness, Injury severity, Clustering analysis, Imbalanced data, Classification and Regression Tree (CART)

## Abstract

**Background:**

Fatigue and drowsiness accidents are more likely to cause serious injuries and fatalities than other accidents. Statistics revealed that 20 to 40 percent of traffic accidents in Iran are due to drivers' fatigue. This study identified the most important factors affecting driver injuries in fatigue and drowsiness accidents.

**Methods:**

The Classification and Regression Tree method (CART) was applied 11,392 drivers were in-volved in fatigue and drowsiness accidents in three provinces of Iran, over the 7 years from 2011-2018. A two-level target variable was used to increase the accuracy of the model. First, dataset in each of three provinces was classified into homogeneous clusters using a two-step clus-tering algorithm. Oversampling method was used for imbalanced accident severity datasets. Then, classification was improved by boosting method.

**Results:**

The classification tree reveals that the month, time of day, collision type, and vehicle type were common factors. Also, driver's age was important in female drivers cluster; the geometry of the place and seat belt/helmet usage were important in urban roads cluster; and area type, road type, road direction, and vehicle factor were important in rural roads cluster. Also, the combination of the CART algorithm with oversampling and boosting increased the accuracy of the models.

**Conclusions:**

The analysis results revealed motorcycles, lack of using a helmet or seat belt, curvy roads, roads with two-way undivided and one-way movement direction increased the injury and death of drivers. Collision with fixed object, run-off-road, overturning, falling, and defective vehicles increased the severity of accidents. Female drivers older than 44 years old have a higher probability of fatality. Identifying the factors affecting the severity of driver injuries in such accidents in each province could assist in determining engineering countermeasures and training educational programs to mitigate these crash severities.

## Introduction

Fatigue and drowsiness have caused countless accidents worldwide.^1^ Drowsiness and fatigue in drivers have been recognized as an important factor causing severe casualties in traffic accidents.^2-5^ Statistics revealed that 20 to 40 percent of traffic accidents in Iran are due to drivers' fatigue.^6^ The impact of fatigue and drowsiness on drivers could be more severe consequences due to the lack of avoidance or corrective action, poor speed control, and slower reaction time.^7,8^ The U.S. National Highway Traffic Safety Administration (NHTSA) estimated that 56,000 drowsiness accidents occur annually, resulting in 1,550 fatalities and 40,000 injuries.^9^ In Australia, fatigue accounted for 15% of heavy vehicle fatal crashes and 10% of total injury crashes, incurring more than $250 million costs.^10^

Severe injuries and fatalities cost much more than light injuries. A single-vehicle crash in which running of the road occurs with no signs of braking or no attempt to prevent the vehicle from colliding with obstacles has been identified as common outcomes of fatigue and drowsiness accidents.^11-14^ Zhang et al.^15^ recognized that male drivers, truck drivers, driving during midnight to dawn and morning peak hour increase the likelihood of fatigue accidents but have no significant effect on the severity of causalities. Moreover, driving at night on the streets without lighting causes drowsiness accidents and severe casualties. Also, some factors such as driving with street lights at night, driving on slippery roads, weekends, unsafe vehicle conditions and less experienced drivers increased the likelihood of causing severe causalities without significant effect on fatigue accidents.

Most studies identified fatigue and drowsiness driving as the major cause of traffic accidents on highways and high speed zones.^15-18^ However, Zhang et al.^15^ conducted that drivers have a lower chance of causing casualties on expresses due to the high quality and better road conditions. Filtness et al.^19^ revealed that driver drowsiness is not restricted to high speed, motorway driving and 41% of all fatigue and drowsiness crashes are represented on the road with low speed. Many drivers also reported having experienced fatigue and drowsiness crashes on low speed road and this kind of accident (regardless of speed zone) are most common when commuting to and from work. Fatigue and drowsiness crashes on low speed roads are important because they occur in densely populated areas, exposing more people to risk and severe consequences.^20^ For this reason, such accidents on low-speed roads require special attention.^19^

From the standpoint of analytic methods, various regression type models have been used in fatigue and drowsiness accidents.^15,19,21-24^ In regression modelling, the relationships between dependent and independent variables should be defined before modelling, also the model estimation will cause erroneous inferences in case the assumptions do not hold.^25^ Some algorithms such as ANN and SVM also have a good ability to predict and classify data, but they cannot provide a proper interpretation of the outputs for analysts and look like a black box difficult to interpret and understand individualized feedback to analysts. To defeat this limitation, classification and regression tree (CART) has been widely utilized to analyze traffic safety.^25,26^ Since traffic crash data occur due to the simultaneous influence of several effective factors, they are usually heterogeneous.^27^ In data mining techniques, clustering is the process of partitioning a set of data into different homogeneous clusters. Several research has employed the clustering techniques to segment crash data into different homogeneous clusters.^28-31^ Another issue with traffic accident data is imbalance, where the number of instances of different classes of the target variable are not equal. If the data imbalance problem is not taken into account, then the performance of classification algorithm would degrade.^32^ In the current study, the resampling technique is used to solve the problem of data imbalance. Moreover, Some researchers have suggested to convert the multi-class target variables into two-class target variables, which increases the prediction accuracy.^33-36^ The boosting algorithm is also used to increase the accuracy of the CART model. Combining the CART algorithm with boosting helps reduce imbalance and variance.^37^

A review of the literature reveals that various factors, including human, vehicle, road, and environment can affect injury severity, fatigue and drowsiness accidents.^15,19,20,38-42^ Therefore, the current study aimed to investigate the factors affecting driver injury severity of fatigue and drowsiness accidents through a clustering approach in three provinces of Iran with different geographical, cultural and climatic locations (the Tehran province (the capital of Iran), Mazandaran province (the north of Iran), and the Fars province (the southwest of Iran)). The two-step clustering, over-sampling and the classification and regression tree (CART) method were combined so as to better identify the important factors. In addition, the classifications were improved by boosting algorithm.

Extracting the important factors affecting driver injury severity in such way that similar fatigue and drowsiness accidents are grouped into separate clusters by their levels of contributory factors would help to priority safety countermeasures, educational programs, and enforcement measures, and future research.

## Methods 

In this study, classification and regression tree was used to investigate the important factors affecting driver injury severity of fatigue and drowsiness accidents in three provinces of Iran.This section provides a summary of this model. In the rest of this section, a two-step clustering was used to divide data into homogeneous clusters, the over-sampling method was used to treat the imbalanced structure of the dataset and investigate whether the accuracy of CART models could be improved using the amplification algorithm.

**Two-step clustering algorithm**

The present study used the two-step clustering algorithm proposed by Chiu et al.^43^ to cluster fatigue and drowsiness data. This method of clustering has some advantages, including its ability to cluster data based on any form of data measurement (continuous and categorical variables) at the same time, work well with large data sets, automatically determine the number of clusters and identify the importance of each item in the cluster solution.^43^

The two-step clustering consists of two distinct stages. In the first step of this algorithm, after reviewing all database records and identifying similar sets of records, the data are classified into pre-clusters. Second, the pre-clusters are used as input and the standard hierarchical clustering algorithm is applied on the pre-clusters. In this step, a range of solutions are produced with different number of clusters and then it can automatically determine the optimal number of clusters by comparing the Bayesian Information Criterion (BIC) across different clustering solutions.^44^

**Balancing**

Imbalancing in database occurs when there is a significant difference between the numbers of samples belonging to different classes of the target variable. If the data imbalance is not treated, the classification model may be biased toward the majority instances. Resampling is one of the prevalent methods (in dealing with the class imbalance problem) to solve the problem of imbalance among classes, which alters the class distribution of samples until the minority class is well demonstrated in the training data.^45^ The data balancing technique applies to the training data (70% of data).

The dataset of fatigue and drowsiness accidents used in this study has imbalanced classes with only 0.99%, 2.4%, and 1.4% of driver fatalities in Tehran, Fars, and Mazandaran. To overcome imbalanced classes, the over-sampling method is applied. In this method, if the number of classes was C, first, the classes are sorted in ascending order based on the size of their samples. Finally, the samples of class 1 to C-1 are randomly selected to the majority class sample (nC).

**CART algorithm**

Classification and regression tree (CART) was used in the current study to identify the factors affecting the driver injuries severity in the fatigue and drowsiness accidents in each cluster. This method was developed by Breiman et al.^37^ In this study seventy, percent of the data were randomly assigned to train and remaining data was allocated to the test.

Tree growing starts at the top of the tree, which locates all the training dataset. Then, a branch is created on the top of tree based on the variable that provides the highest homogeneity in each branch. Then, the top of the tree will be divided into two subsets by an independent variable that leads to the most significant improvement in the purity of two subsets. This process goes on and on for each child node until all observations in each terminal node or ‘‘leaf’’ have the greatest possible homogeneity. In the CART model, to achieve the optimal tree, the tree pruning operation is performed using the misclassification cost method. Besides, one of the most important advantages of the decision and regression tree is determining the importance of variables. 

**Crash data**

The data of fatigue and drowsiness accidents that occurred in Tehran, Fars and Mazandaran provinces during 2011-2018 were collected from the Traffic Police Accident Database.

To identify the factors affecting the driver injury severity in fatigue and drowsiness accidents, eighteen independent variables were analyzed. The dependent variable was the driver injury severity, which are divided into three categories: no-injury, injury and fatality. Finally, after clearing the database, 5568, 4072 and 1758 crash data remained for Tehran, Fars, and Mazandaran provinces. Table 1 presents the study variables and subcategories of each variable in the current study for three provinces (Tehran, Fars, and Mazandaran).

**Table 1:** Variable description in each province.

Variable	Levels	Frequency %		
		Tehran	Fars	Mazandaran
**Drivers’ gender**	Male	93.7	95	94
	Female	6.3	5	6
**Drivers’ age**	<25	10.5	10.3	11.8
	25-44	63.6	67.7	66.4
	>44	25.9	22	21.7
**Vehicle type**	Auto	68.8	63	69.3
	Pick	7.6	9.8	10.2
	Truck	19.7	22.8	14.9
	Motorcycle	3.9	4.4	5.6
**Vehicle defect**	Yes	6.8	35.7	23.8
	No	93.2	64.3	76.2
**Restraint use**	Used	16.1	17.4	16.4
	Not used	5.5	3	3.3
	Unknown	78.3	79.6	80.3
**Terrain**	Rolling	3	1.4	1.5
	Level	91.3	96.7	92.5
	Mountainous	5.8	2	6
**Roadway geometry**	Straight and level	11.4	3.6	4
	Straight and grade	84	89.7	88
	Curve and level	2.7	1.9	3.1
	Curve and grade	1.9	4.8	4.9
**Road type**	Freeway	18.4	21.91	12.89
	Highway	34.36	22.01	2.97
	Major Road	13.89	36.69	52.03
	Minor road	1.79	10.02	7.93
	Major street	28.24	8.35	18.2
	Minor street	3.23	0.52	1.48
	Direct road	0.09	1.5	4.51
**Shoulder type**	Paved	15.6	33	10.9
	Stabilized gravel	7.8	29.5	26.4
	None	76.6	37.5	62.7
**Road configuration**	Two-Way, Not Divided	13.3	24.1	31.1
	Two-Way, Divided	61.8	17	48.9
	One-Way	25	58.9	20
**Land use**	Non-Residential	41.9	63.2	22.8
	Residential	58.1	36.8	77.2
**Area type**	Suburban	35	88	64.6
	Urban	65	12	35.4
**Traffic control**	Have control	46.2	13.3	28.2
	No control	15.4	68.9	50.4
	Unknown	38.4	17.8	21.5
**Collision type**	Fixed object collision	17.8	7.9	13.9
	Collision with motorcycle	7.4	9.2	14.2
	Two vehicle collision	63.2	44.4	60.2
	Running off	3.8	16.4	2.8
	Overturning	7.8	22.1	8.9
**Lighting condition**	Day light	53.7	60	64
	Dark	41	35.6	30.1
	Dusk/dawn	5.3	4.5	5.9
**Time-of-the-day**	24-02	9.8	7.2	7.2
	02-04	10.5	7.2	6.4
	04-06	11.1	6.7	5.2
**Time-of-the-day**	06-08	12.6	10	8
	08-10	8.3	8.4	6.5
	10-12	5.8	8.1	8
	12-14	6.5	9.6	9.3
	14-16	8.6	9.9	10.9
	16-18	7.4	8.2	11.2
	18-20	6	8.4	9.9
	20-22	5.6	7.4	9.2
	22-24	7.8	8.9	8.3
**Day-of-the-week**	Saturday	13.2	14.2	13.1
	Sunday	15	14.1	13.1
	Monday	14.5	13.4	12.7
	Tuesday	13.9	14.3	14.1
	Wednesday	14.1	13.7	16.1
	Thursday	15.8	15.7	15.3
	Friday	13.5	14.6	15.6
**Month-of-year**	April	7.9	9.2	9.7
	May	10.3	8.6	7.2
	June	11.2	9.7	7
	July	10.9	10.1	10.4
	August	9.7	12.9	7.6
	September	9.8	12.5	8.9
	October	7.7	8.5	6.9
	November	7.4	9.3	11
	December	6.6	5.3	9.2
	January	6.1	5	8.2
	February	5.9	4.2	8.9
	March	7.3	3.9	5

## Results

**Reducing the problem of multi-class prediction into a set of two-class prediction models**

In this study, the target variable (driver injury severity) is divided into three categories: no-injury, injury, and fatality. Some researchers have suggested to convert the multi-class target variables into two-class target variables, which increases the prediction accuracy.^33-36^ In this study, according to Delen et al.^34^ and Dissanayake and lu,^35^ instead of presenting a model to predict the driver injury severity in which the response variable (target) has three not-injury, injury, and fatality levels, four models were proposed with two-level response variables, zero and one. The classification of the four models is summarized in Table 2.

**Table 2:** Graphical representation of two-class target variable model configurations.

Model Label	No-injury		Injury		Fatality
1.1		At most injury			Fatality
1.2	No-injury		Injury		
2.1	No-injury			At least injury	
2.2			Injury		Fatality

**Data mining framework**

**Figure 1:**
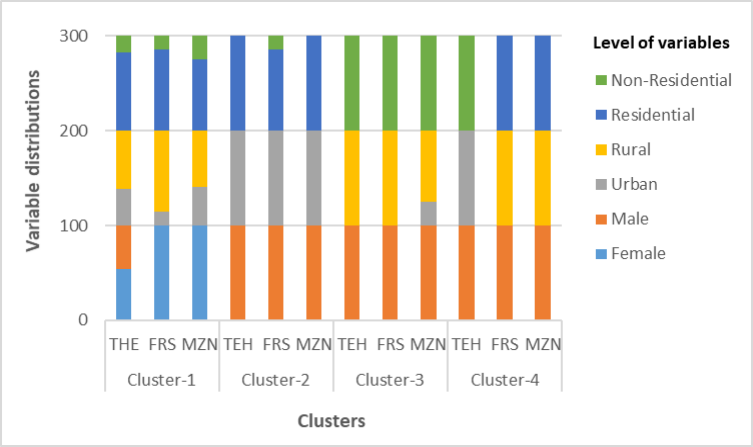
Variables and univariate distributions in each province’s cluster for models 1.1, 1.2 and 2.1 (THE = Tehran, FRSA = Fars, MZN = Mazandaran)

**Figure 2:**
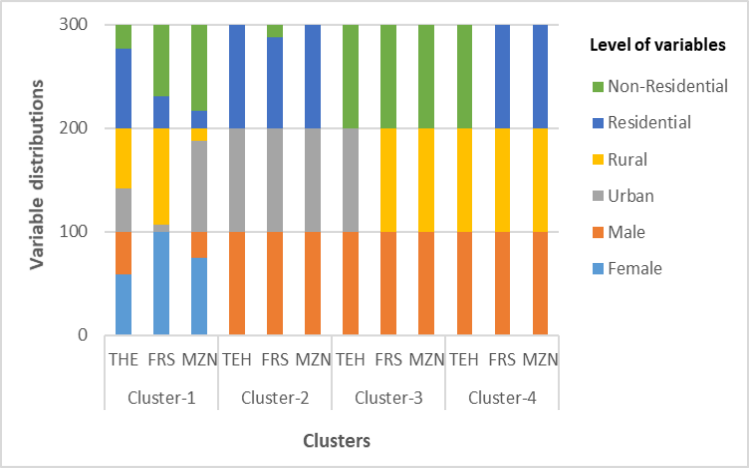
Variables and univariate distributions in each province’s cluster for model 2.2 (THE = Tehran, FRSA = Fars, MZN = Mazandaran)

The first step in data mining framework was to apply two-step clustering to fatigue and drowsiness data. The clustering of fatigue and drowsiness accidents was performed using all the variables presented in Table 1. The optimal cluster number for Tehran and Mazandaran provinces was k=4 with a silhouette coefficient of 0.9. The optimal cluster number of Fars province was also k = 4 with a silhouette coefficient of 0.8. If the silhouette coefficient for the cluster analysis ranges from 0.71 to 1, it can be stated that the algorithm was able to discover a "strong" cluster structure among the data.^46^ Characteristics of clusters were determined based on their variable distributions. Variables and univariate distributions in each province’s cluster are for 3 models (1.1, 1.2, and 2.1) in Figure 1 and one model (2.2) in Figure 2.

The four clusters for three models (1.1, 1.2, and 2.1) were named and presented in Table 3 . The name of clusters in model 2.2 for Tehran and Fars provinces was the same as other models. In Mazandaran province, clusters 2 and 4 in model 2.2 were named similar to clusters 2 and 4 in models 1.1, 1.2, and 2.1. However, in cluster 3, 100% occurrence of fatigue and drowsiness accidents was for male drivers in non-residential land uses of rural areas. Therefore, this cluster is referred in model 2.2 as "fatigue and drowsiness accidents for male drivers in non-residential, rural areas". Also, cluster 1 was the only cluster that had female drivers in addition to male drivers. Therefore, this cluster is referred as "fatigue and drowsiness accidents for female and male drivers".

**Table 3:** Cluster descriptions for models 1.1, 1.2, and 2.1.

Tehran	Fars	Mazandaran
fatigue and drowsiness accidents for female and male drivers	fatigue and drowsiness accidents for female drivers	fatigue and drowsiness accidents for fe-male drivers
fatigue and drowsiness accidents for male drivers in residential, urban areas	fatigue and drowsiness accidents for male drivers on urban roads	fatigue and drowsiness accidents for male drivers in urban, residential land uses
fatigue and drowsiness accidents for male drivers in non-residential, rural areas	fatigue and drowsiness accidents for male drivers in non-residential, rural areas	fatigue and drowsiness accidents for male drivers in non-residential land uses
fatigue and drowsiness accidents for male drivers in non-residential, urban areas	fatigue and drowsiness accidents for male drivers in residential, rural areas	fatigue and drowsiness accidents for male drivers in rural, residential land uses

**Figure 3:**
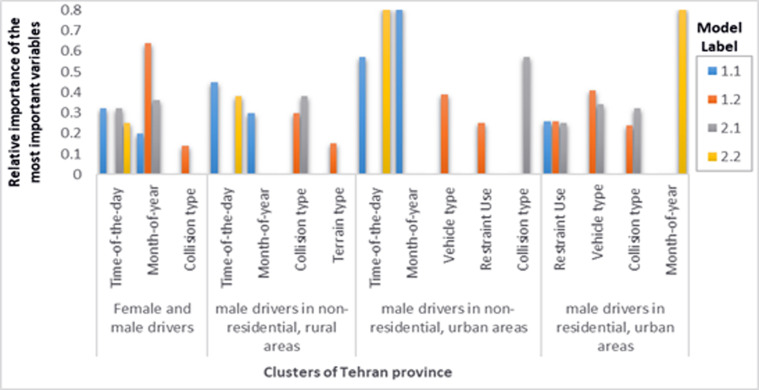
Relative importance of the most important variables in clusters of Tehran province

**Figure 4:**
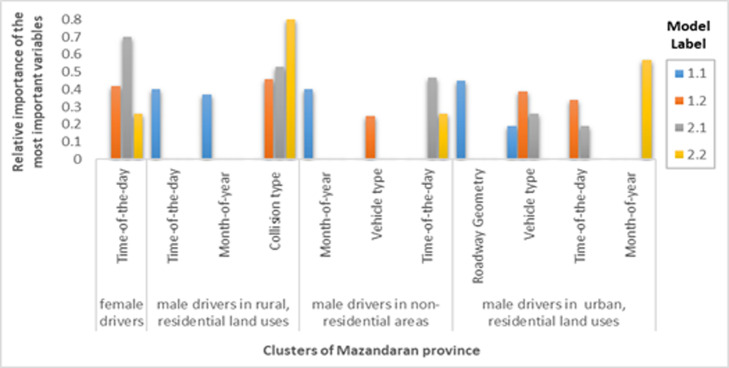
Relative importance of the most important variables in clusters of Mazandaran province

**Figure 5:**
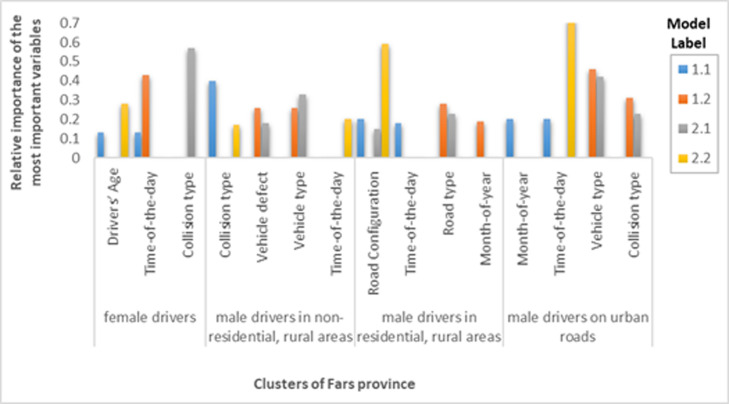
Relative importance of the most important variables in clusters of Fars province

After grouping the data into four homogeneous clusters, the driver injury severity was balanced using the over-sampling method. Finally, after grouping and balancing the training set, the most important independent variables of each cluster were identified using the CART model. Figure 3-5 show the most important variables of Tehran, Mazandaran, and Fars provinces.

In the rest of this section, the increased probability of driver injuries (models 1.2) and at least driver injuries (models 2.1) and then the increased probability of driver fatality (models 1.1 and 2.2) are examined by identifying the most important variables in fatigue and drowsiness accidents in three clusters as follows:

- Cluster 1: ‘fatigue and drowsiness accidents for male drivers on urban roads.’

- Cluster 2: ‘fatigue and drowsiness accidents for male drivers on rural roads.’

- Cluster 3: ‘fatigue and drowsiness accidents for female drivers.’

**The Probability of injury (model 1.2) and at least injury (model 2.1)**

***Fatigue and drowsiness for male drivers on urban roads (cluster 1)***

In the residential and non-residential areas of Tehran province, the probability of driver injury and severity injury increases if the driver did not use a helmet or seat belt. Also, in the residential areas, the injury severity increases for motorcyclists and vehicle collision with a fixed object, run-off-road, overturning, and falling. However, in the non-residential areas, pickup trucks and motorcycles are found to increase the probability of injury severity for drivers. Besides, vehicle collision with a motorcycle, vehicle collision with a fixed object, overturning, and falling increase the probability of at least injury and fatality for drivers.

In the residential areas of Mazandaran province, the probability of no injury to the motorcyclists is very low, the probability of no injury to the drives is reduced between 8 and 10 pm and 8 am to 12 noon. Also, in 6-8 pm, the probability of drivers being injured increases, and the probability of at least injury and fatality increases at 2-4 am and 6-8 am.

In the residential and non-residential areas of Fars province, driver injury severity increases for motorcyclists, collision of a vehicle with a fixed object, run-off-road, overturning, and falling.

***Fatigue and drowsiness for male drivers on rural roads (cluster 2)***

In the non-residential areas of Tehran province, the probability of drivers being injured increases for the run-off-road collision, fixed object collision, two vehicle collision, and in rolling and mountainous areas. Besides, at least injury and fatality of drivers increases for overturning, falling, and vehicle collision with a motorcycle.

In the residential areas of Mazandaran province, the probability of severe injuries increases in run-off-road collision, overturning and falling collision, vehicle collision with a fixed object, and vehicle collision with a motorcycle.

In the residential areas of Fars province, on all types of roads (except freeways and highways), the probability of not injured drivers is very low. In addition, on this type of roads, the probability of at least injury and fatality for drivers increases if the direction of movement is two-way undivided and one-way. Also, in January, April, May, June, July, and August, the probability of drivers being injured increases. However, in the non-residential areas of Fars province, the motorcycle vehicle and if the vehicle was defective would increase at least injury and fatality of drivers.

***Fatigue and drowsiness for female drivers (cluster 3)***

Time of day variable was common among three provinces. The probability of at least injury and fatality increases in Tehran province from 12 midnight to 10 am and from 2 pm to 8 pm, and in Mazandaran province at 2-4 pm. Also, in Fars province, female drivers are more likely to be injured from 10 pm to 12 midnight.

In addition, in Tehran province, the probability of injury or at least injury and fatality of female drivers increases in January, February, June, August, September, and November. Besides, the probability of injury to female drivers increases for run-off-road collision and vehicle collision with a motorcycle. However, the probability of at least injury and fatality to female drivers increases for the fixed object collision, overturning and falling collision, and run-off-road collision in Fars province.

**The probability of fatality (models 1.1 and 2.2)**

***Fatigue and drowsiness for male drivers on urban roads (cluster 1)***

In residential and non-residential areas of Tehran province, the probability of drivers' fatality increases in January, February, May, June, and October. Furthermore, the probability of drivers' fatality increases in March and September in the residential areas and in April in the non-residential areas. Also, the probability of drivers' fatality increases in cases drivers did not use a helmet or seat belt in the residential areas, and from 4 am to 10 am and from 4 pm to 6 pm in the non-residential areas.

In the residential areas of Mazandaran province, the probability of fatality increases in trucks and motorcycles, on the curvy roads, and in March, July, and October.

In residential and non-residential areas of Fars province, the probability of drivers' fatality increases in June from 4 to 6 am.

***Fatigue and drowsiness for male drivers on rural roads (cluster 2)***

In the non-residential areas of Tehran province, the probability of drivers' fatality increases from 10 pm to 12 midnight, at 2-4 am, from 8 am to 2 pm, at 4-6 pm and also in February, March, May, June, July, August, and September.

In Mazandaran province, the probability of drivers' fatality in the residential areas increases at 2-6 pm and from 12 midnight to 2 am, however in the non-residential areas increases at 2-4 pm, 6-8 pm, and from 10 pm to 12 midnight. Besides, the probability of drivers' fatality in the residential areas increases in January, July, August, and October.

In Fars province, the probability of drivers' fatality in the residential areas increases at 2-10 am and 4-6 pm, but in the non-residential areas increases at 2-8 am, from 12 noon to 4 pm, and at 8-10 pm. In addition, the probability of drivers' fatality in the residential areas increases on two-way undivided roads and the non-residential areas increases for two vehicle collision, fixed object collision, and overturning and falling collision.

***Fatigue and drowsiness for female drivers (cluster 3)***

Time of day variable was common among three provinces. The probability of female drivers' fatality in Tehran province increases at 4-6 pm and from 12 midnight to 4 am, in Mazandaran province increases from 12 midnight to 2 am and at 4-6 am, and in Fars province increases from 8 am to 10 am. Also, the probability of female drivers' fatality in Tehran province increases in January, February, and June, and in Fars province increases for over 44 years old.

According to Table 4, in addition to the high overall accuracy of the models, the majority class (class 1) and the minority class (class 2) have high accuracy when combining the CART model with the oversampling method. Also, combining the boosting algorithm with the oversampling method produces better results. This study indicated that the boosting method could be effective in handling imbalanced data when combined with the oversampling method.

**Table 4:** Prediction accuracy by treatments.

Model Label	Province	Cluster description	Over-sampling	Over-sampling + Boosting				
			Accuracy			Accuracy		
			Overall%	class 1 %	class 2 %	Overall%	class 1 %	class 2 %
1.1	Mazandaran	male drivers in urban, resi-dential land uses	94.78	100	90.55	100	100	100
		male drivers in rural, residen-tial land uses	94.56	100	90.18	100	100	100
		male drivers in non-residential land uses	97.18	100	94.65	100	100	100
	Fars	female drivers	100	100	100	100	100	100
		male drivers in residential, rural areas	97.23	100	94.74	100	100	100
		male drivers in non-residential, rural areas	72.53	77.29	69.18	85.86	87.6	84.25
		male drivers on urban roads	99.84	100	99.67	100	100	100
	Tehran	female and male drivers	97.23	100	94.73	100	100	100
		male drivers in residential, urban areas	97.81	100	95.81	97.81	100	95.81
		male drivers in non-residential, urban areas	91.92	100	86.08	99.78	100	99.57
		male drivers in non-residential, rural areas	81.57	100	73.04	97.42	100	95.1
1.2	Mazandaran	female and male drivers	91.78	100	85.88	100	100	100
		male drivers in urban, resi-dential land uses	69.54	67.59	71.98	97.94	100	96.04
		male drivers in rural, residen-tial land uses	73.1	72.86	73.34	93.69	97.26	90.64
		male drivers in non-residential land uses	73.72	71.98	75.75	95.6	97.11	94.2
	Fars	female drivers	60.85	57.63	71.1	85.49	87.77	83.5
		male drivers in residential, rural areas	76.7	74.79	78.91	82.21	81.86	80.57
		male drivers in non-residential, rural areas	66.18	67.89	64.76	70.41	70.41	70.4
		male drivers on urban roads	74.45	79.18	72.5	94.44	99.01	90.65
	Tehran	female and male drivers	72.73	81.7	67.75	95.92	100	92.45
		male drivers in residential, urban areas	80	78.12	82.16	84.23	85.64	82.93
		male drivers in non-residential, urban areas	78.74	82.24	75.84	93.75	96.36	91.41
		male drivers in non-residential, rural areas	68.71	71.24	66.69	77.98	78.9	77.13

## Discussion

As provided in Figure 3-5 among the important variables that increase the driver injury severity in fatigue and drowsiness accidents, time of day, month, collision type, and vehicle type were common among different clusters of provinces. The time of day and month that affect the severity of drivers' injuries was different in each province cluster, which can be attributed to the cultural, geographical, climate, and environmental differences, etc. Fatigue accidents were common in most different clusters of all three provinces for collision with fixed object, run-off-road, overturning, and falling, and increased the severity of accidents. This is in line with other studies.^11-13,14^ For instance, Sagberg et al. have indicated that more than 34% of fatigue accidents were run-off-road.^11^ Moreover, Radun et al. showed that more than 80% of fatigue accidents in Finland are of single-vehicle type.^13^ Results of the current study indicated that motorcycles were more injured and died in fatigue and drowsiness accidents. This could be due to the relatively small size with a powerful engine and high speed, lack of protection for the riders, complexity in manoeuvrability, and low motorcycle stability.^38^

Previous studies in other fields have shown that not using a helmet and seat belt plays a significant role in increasing the severity of accident injuries.^39-41^ This is similar to the results of this study, in the rural roads of Tehran province, lack of using a helmet or seat belt increases the injury and death of drivers.

Results also showed that defective vehicles lead to severe casualties. This result has also been shown in Zhang et al.^15^ where they reported that unsafe vehicles lead to severe casualties, because it is difficult to control such vehicles in the event of an accident, and their vehicles indicated the low safety awareness of their drivers. The results of this study also indicated that fatigue and drowsiness accidents on freeways and highways are less likely to cause severe casualties. Moreover, results revealed that female drivers older than 44 years old in Fars province have a higher probability of fatality. Whereas, most studies indicated that due to the age related factors, most young drivers are involved in fatigue and drowsiness accidents,^13,42^ but older drivers couldn’t handle the situation due to lack of rapid response in emergencies and weak body physics, therefore, older drivers were more likely to involve severe casualties than young drivers. On the other hand, our results showed that drivers are more likely to be injured and died on the curvy roads in the residential urban areas of Mazandaran province, and roads with two-way undivided and one-way movement direction in Fars province. This is in line with studies that have shown that driver drowsiness is not limited to high speeds and these kinds of crashes on low-speed roads are important because they occur in densely populated areas, exposing more people to risk and severe consequences.^19,20^ In addition, in mountainous non-residential areas of rural roads in Tehran province, drivers are more likely to be injured. One of the reasons is that the driver has to focus more on the road, which can cause driver fatigue. Moreover, high speed, delay in proper reaction, and driver's inability to control the vehicle in this situation can cause serious injuries to drivers.

## Conclusion

The present study used a data mining framework. First, fatigue and drowsiness crash data were divided into homogeneous groups using the clustering analysis based on land use, area type, and driver gender variables. Next, the oversampling technique was used to balance the driver's injury severity in fatigue and drowsiness accidents. Then, Classification and regression tree is applied to identify the most important variables affecting driver injuries in fatigue and drowsiness accidents. Finally, the boosting algorithm was used to increase modelling accuracy. Among the important variables that increase the driver injury severity in fatigue and drowsiness accidents, time of day, month, collision type, and vehicle type were common among different clusters of provinces. Besides, the geometry of the place and the status of using the seat belt/helmet were important factors in male drivers on urban roads (cluster 1); the area type, road type, road direction, and vehicle factor were important factors in male drivers on rural roads (cluster 2); driver's age was important factor in female drivers (cluster 3). According to the results, motorcycles, lack of using a helmet or seat belt, curvy roads, roads with two-way undivided and one-way movement direction increased the injury and death of drivers. Collision with fixed object, run-off-road, overturning, falling, and defective vehicles increased the severity of accidents. These issues might be addressed by imposing strict rules and hefty fines If the seat belts and helmets are not used. Besides, two-way divided roads and Proper use of guardrails and rumble strips can reduce the drivers' injury severity in these accidents. The results indicated that defective vehicles increased the severity of accidents. Therefore, the drivers' awareness about their car conditions should be raised. Female drivers older than 44 years old have a higher probability of fatality. Drivers might be informed about these issues by providing public awareness campaigns. 

Also, combining the CART algorithm with boosting algorithm and the oversampling method produces better results than just with oversampling method. It can be concluded that the combined use of clustering, balancing, CART algorithm and boosting could be useful in identifying the factors affecting driver injury and prioritizing safety countermeasures and training programs proportional to each group of fatigue and drowsiness accidents. 

Future studies can identify the most important factors through questionnaires among different groups of drivers or use traffic accident insurance data and compare the results with the current study. Finally, comparing the performance of the k-means and Latent Class algorithms, for clustering crash data, with performance "two-step" and other methods for overcoming the data imbalance problem might be a good topic for future research. This might help to provide the most efficient algorithm for clustering and balancing the driver's injury severity in fatigue and drowsiness accidents.
